# Transcriptomic divergence of the *Rheum palmatum* complex derived from top-geoherb and non-geoherb areas provides the insights into geoherbalism properties of rhubarb

**DOI:** 10.1186/s12864-024-10142-3

**Published:** 2024-02-26

**Authors:** Tao Zhou, Yadi Tang, Lipan Zhou, Jie Liu, Yang Pu, Fan Jiang, Jian Wang, Xumei Wang

**Affiliations:** School of Pharmacy, Xi’an Jiaotong University, Xi’an, 710061 China

**Keywords:** *Rheum palmatum* complex, Transcriptome, Geoherbalism, Gene expression profiles, Positive selection

## Abstract

Geoherb usually represents high-quality medicinal herbs with better clinical therapeutic effects, and elucidating the geoherbalism is essential for the quality improvement of traditional Chinese Medicine. However, few researches were conducted to clarify the geoherbalism based on a large scale of transcriptomics. In the present study, we compared the transcriptomes of *Rheum palmatum* complex derived from top-geoherb and non-geoherb areas to show the geoherbalism properties of rhubarb. A total of 412.32 Gb clean reads were obtained with unigene numbers of 100,615 after assembly. Based on the obtained transcriptome datasets, key enzyme-encoding genes involved in the anthraquinones biosynthesis were also obtained. We also found that 21 anthraquinone-related unigenes were differentially expressed between two different groups, and some of these DEGs were correlated to the content accumulation of five free anthraquinones, indicating that the gene expression profiles may promote the geoherbalism formation of rhubarb. In addition, the selective pressure analyses indicated that most paired orthologous genes between these two groups were subject to negative selection, and only a low proportion of orthologs under positive selection were detected. Functional annotation analyses indicated that these positive-selected genes related to the functions such as gene expression, substance transport, stress response and metabolism, indicating that discrepant environment also enhanced the formation of geoherbalism. Our study not only provided insights for the genetic mechanism of geoherbalism of rhubarb, but also laid more genetic cues for the future rhubarb germplasms improvement and utilization.

**Supplementary Information:**

The online version contains supplementary material available at 10.1186/s12864-024-10142-3.

## Introduction

Geoherb (Daodi yaocai in Chinese) refers to the traditional Chinese Medicine (TCM) from the specific geographic areas, which generally represents high-quality medicinal herbs with better clinical therapeutic effects [1]. From a biological point of view, different individuals/populations derived from the same species can be divided into either top-geoherbs or non-geoherbs based on their chemical constituents [2]. Conventionally, the generic term of various merits possessed by top-geoherbs is called as geoherbalism which has formed throughout a long historical process of usage in TCM industry [3]. Clarifying the causes of geoherbalism not only help to identify the quality characteristics of top-geoherbs, but also provide more insights into the germplasm cultivation and quality improvement of TCMs. In the past decades, although geoherbalism of the TCMs had catered to the researchers’ interests, studies have been conducted on the limited medicinal materials such as Huangqin (Scutellariae Radix) and Danggui (Angelicae Sinensis Radix) to evaluate the geoherbalism [1, 4]. Therefore, the formation mechanism of geoherbalism should be elucidated for important bulk medicinal materials and then lay the foundation for the medicinal plant cultivation and medicine utilization.

With the increasing demand of TCM, more and more researchers have paid attention on the quality formation of TCMs and tried to explore quality differences of TCMs [5–7]. The quality difference of top-geoherbs and non-geoherbs is mainly determined by the category and quantity of specific metabolites which were used to treat disease, and the metabolite diversity is usually derived from the interaction between minor-polygenes and discrepant environment [2]. Huang et al. [3] also mentioned that the ecological and genetic factors are essential for the inference of mechanism of geoherbalism. Recently, studies have showed that the ecological factors greatly influenced the quality of TCMs and provided the insights for the formation of geoherbalism [2, 8–10]. In addition, the available researches showed that obvious geoherbalism was derived from the genetic differentiation between top-geoherbs and non-geoherbs [11, 12]. However, it was widely recognized that the biosynthesis of metabolites for medicinal plants may be regulated by the specific key enzyme genes. Especially, the characters and expression profiles of such structural enzyme genes for medicinal plants under the different environment also had profound impact on the accumulation and production of specific secondary metabolites [13]. Therefore, showing the variations and expression profiles of genes related to the biosynthesis of effective compounds for the medicinal plants may not only help us to elucidate the genetic causes for the quality difference between top-geoherbs and non-geoherbs, but also provide more cues for the geoherbalism of medicinal materials.

Rhubarb, derived from the dried roots and rhizomes of any species of *Rheum officinale* Baill., *R. palmatum* Linn., or *R. tanguticum* (Maxim. ex Regel) Maxim. ex Balf. (Polygonaceae), is one of the main exports of TCM. As a commonly used TCM, rhubarb was first documented in Shennong Bencao Jing (Shennong’s Classic of Materia Medica; 200–300 AD) with the efficacies such as cooling blood, detoxification, removal of blood stasis, removing dampness, abating jaundice, etc. [14]. The major medicinally active compounds of rhubarb are anthraquinones, which were usually used for the quality evaluation of rhubarb in the Pharmacopoeia of the People’s Republic of China [14]. The previous genetic and morphological data showed that three abovementioned source plant species of rhubarb can be regarded as one species (*R. palmatum* complex) [15, 16]. Conventionally, rhubarb collected from Qinghai, Gansu, and Sichuan provinces (in or near the QTP and the Hengduan Mountains) are defined as top-geoherbs while others collected from eastern areas of China are classified to be non-geoherbs with inferior quality. Our previous study has found that there were obvious genetic and climatic divergences between top-geoherbs and non-geoherbs [11], indicating that the formation of geoherbalism is correlated to genetic and ecological factors. However, the characters and expression profiles of key enzyme genes related to the biosynthesis of medicinally active ingredients in rhubarb from top-geoherbs and others are still unclear. With the advent of high throughput sequencing in recent years, it has become comparatively easy to sequence the transcriptome of medicinal plants and identify the structural enzyme genes involved in the metabolite biosynthesis and the expression profiles.

In this study, we investigated the transcriptomic divergence between the traditionally recognized top-geoherbs and non-geoherbs regions for *R. palmatum* complex. We try to address whether there has been a transcriptomic divergence between the top-geoherbs and non-geoherbs of rhubarb, and whether the expression profiles of structural enzyme genes involved in the biosynthesis of medicinally active ingredients of rhubarb contributes to the formation of geoherbalism.

## Materials and methods

### Plant materials

We chose 55 samples, which contains 25 typical top-geoherbs and 30 typical non-geoherbs, from Gansu, Qinghai, Sichuan, Shaanxi, Hubei, Henan and Shanxi provinces, China (Table S1) to investigate their transcriptomic divergence. These typical top-geoherb and non-geoherb areas were traditionally recognized and genetically differentiated based on the previous populations structure inference [11]. The source species of all samples were identified by Prof. Xumei Wang at School of Pharmacy, Xi’an Jiaotong University, and the original photos of all samples were shown in Fig. S1. The voucher specimens were deposited in the Herbarium of School of Pharmacy in Xi’an Jiaotong University, Xi’an, China, and the specimen numbers were listed in Table S1. In order to infer the transcriptomic divergences of rhubarb from top-geoherb and non-geoherb areas, three source plants of rhubarb with different leaf morphologies were treat as one species based on previous research [15, 16], and their medicinal parts (roots) were finally used for RNA sequencing. The fresh roots were collected and put into liquid nitrogen immediately for 24–48 h, and then the feezed samples were transferred and stored at -80 °C for total RNA isolation.

### RNA isolation, cDNA library construction and Illumina sequencing

Total RNA of each sample was extracted using the RNeasy Plant Mini Kit (Qiagen, Valencia, CA) according to the steps described in the manufacturer’s protocol. RNA concentration and purity were measured using NanoDrop 2000 (Thermo Fisher Scientific, Wilmington, DE). RNA integrity was assessed using the RNA Nano 6000 Assay Kit of the Agilent Bioanalyzer 2100 system (Agilent Technologies, CA, USA). cDNA libraries were constructed following procedures described by Zhou et al. (2016) and then sequenced using the Illumina X Ten platform (150 bp reads) [17].

### *De novo* assembly and gene annotation

Raw reads produced by Illumina sequencing platform were processed with fastp v. 0.12.6 to remove adaptors, reads with more than 5% of unknown base calls, and low-quality reads (> 20% of the bases with a quality score ≤ 10) [18]. After trimming, the high-quality reads from the top-geoherb and non-geoherb groups were de novo assembled using Trinity v2.5.1 with the default parameters, respectively [19]. After assembly, the resultant transcripts were further processed by CD-HIT v4.6 with a sequence identity threshold of 0.95 to remove redundancies [20]. In order to generate the reference transcriptome dataset for *R. palmatum* complex, the obtained unigenes from top-geoherb and non-geoherb regions were pooled together and further assembled into non-redundant unigenes using the TIGR Gene Indices Clustering (TGICL) tools with the parameters of a 95% similarity and an overlap length of 40 bp [21]. The obtained non-redundant unigenes were searched against the public databases, including NCBI non-redundant protein (Nr), Swiss-Prot, Cluster of Orthologous Group (COG), euKaryotic Ortholog Group (KOG) and Evolutionary Genealogy of Genes: Non-supervised Orthologous Groups (eggNOG), using BLAST with an E-value threshold of 1E-5 [22]. Kyoto Encyclopedia of Genes and Genomes (KEGG) classification was conducted using the KEGG Automatic Annotation Server (KAAS) with an E-value of 1E-10. Protein family (Pfam) alignments were performed using the HMMER v3.0 (http://hmmer.org/) with an E-value of 1E-5, and the Gene Ontology (GO) was classified based on the annotation results of Nr using Blast2GO v2.5 with an E-value of 1E-5 [23].

### Differentially expressed genes (DEGs) between top-geoherb and non-geoherb groups

All the clean reads of samples derived from top-geoherb and non-geoherb groups were mapped to the non-redundant unigenes using Bowtie v2.3 under the default parameters [24]. FPKM (Fragments per Kilobase of transcript per Million mapped reads) values, which commonly used to evaluate the gene expression levels, were estimated by RSEM v1.2.19 with the default parameters [25]. After estimation of the gene expression level of each sample, DEGs between top-geoherb and non-geoherb groups were screened using DESeq2 v1.6.3 [26], and false discovery rate (FDR) < 0.01 and |log2(FoldChange)|> 2 was set as the threshold to evaluate the significance level of differential gene expression. The potential gene functions and candidate metabolic pathways of DEGs were also predicted by searching against the abovementioned public databases, and the statistical enrichment of DEGs in GO terms and KEGG pathways were conducted using ClusterProfile v3.14.0 [27].

### The correlation between the expression levels of differentially expressed enzyme genes and the content of five free anthraquinones

Based on the annotation information and DEGs, the anthraquinone-related genes which showed different expression levels between top-geoherb and non-geoherb groups were identified. In addition, the contents of five free anthraquinones including aloe-emodin, rhein, emodin, chrysophanol and physcion were recovered from our previous study [28]. In order to further clarify the effects of the expression levels of specific genes on the accumulation of anthraquinones, the average FPKM values of differentially expressed key enzyme genes related to anthraquinone biosynthesis and the average contents of free anthraquinones were retrieved for the following correlation analysis. Clustering and correlation analyses were carried out by using cor function of pheatmap package in R v3.5.1, and the correlation degree was evaluated by correlation coefficient values.

### Quantitative real‑time PCR (qRT‑PCR) analysis for the candidate anthraquinone-related genes

In order to validate the accuracy of transcriptome datasets, 15 candidate anthraquinone-related DEGs detected in this study were randomly selected for the qRT-PCR using specific primers (Table S2) designed by Primer 3 [29]. The housekeeping gene actin was used as the internal control for normalization. Total RNA of each sample was isolated using the aforementioned procedures. After removing the genomic DNA, the first-strand cDNA was synthesized using GoldenstarTM RT6 cDNA Synthesis Mix (Beijing Tsngke Biotech Co. Ltd., China) with 2 µL RNA as template. The qPCR was conducted using One step TB Green™ PrimeScript™ RT-PCR Kit (Takara biomedical Technology, China Co. Ltd.), and all the reactions were performed in Agilent MX3000P QPCR Systems (Agilent Technologies, Santa Clara, CA, USA) as follow: 95℃ for 60 s, followed by 40 cycles of 95℃ for 5 s, 60℃ for 10 s, and at 72℃ for 15 s. All sample runs were repeated three times for the consistency, and relative expression levels of genes were calculated using the 2^−ΔΔCt^ method [30].

### Identification of orthologs and estimation of substitution rates between top-geoherb and non-geoherb groups

Open reading frame (ORF) of the unigene datasets from top-geoherb and non-geoherb groups were predicted by the Getorf program with a minimum length of 150 amino acids and translated into peptides [31]. Protein sequences from two groups were used to identify the putative orthologous genes using OrthoMCL [32] with the default parameters. The obtained orthologs were aligned and formatted with ParaAT1.0 under the default parameters [33]. The KaKs_Calculator v2.0 was used to estimate nonsynonymous (Ka), synonymous (Ks) substitution rates, and Ka/Ks ratios of each putative orthologous pair with the YN algorithm [34].

## Results

### Summary statistics of transcriptome sequencing

Fifty-five cDNA libraries were constructed and utilized for the transcriptome sequencing on the Illumina X Ten platform. After sequencing and trimming, high-quality clean reads generated from each library ranged from 21,436,968 to 30,310,981 (Table S3). The Q30 value of each sample was up to 91.26%, and the GC content of each sample ranged from 47.81 to 50.56% (Table S3). The results indicated that these high-quality reads could be used for the subsequent analyses.

### Transcriptome assembly and functional annotation

The clean reads derived from top-geoherb and non-geoherb groups were separately assembled using the de novo assembly strategy by Trinity. After assembly, a total of 427,833 unigenes were obtained for top-geoherb samples with an average length of 462 bp and N50 of 510 bp, and 891,302 unigenes with an average length of 438 bp and N50 of 460 bp were recovered for the non-geoherb samples. After pooling and reassembling pre-assembled unigenes for two groups, 100,615 non-redundant unigenes with an average length of 1,261 bp and N50 of 2,297 bp were retrieved for the functional annotation and DEG analyses. For the non-redundant unigenes, 21,632 (21.50%) had a length > 2,000 bp, 21,192 (21.06%) had a length between 1,000 and 2,000 bp, 17,572 (17.46%) had a length between 500 and 1,000 bp and 10,305 (10.24%) had a length between 300 and 500 bp.

**Fig. 1:**
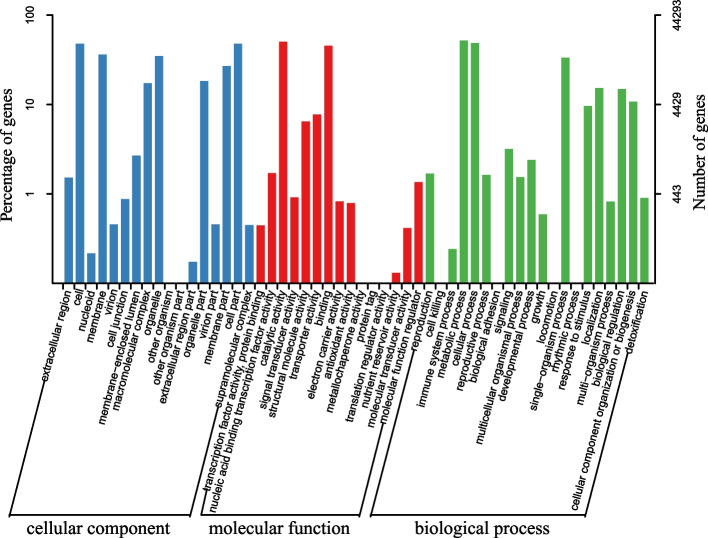
Gene ontology classification of non-redundant unigene sequences from *R. palmatum* complex transcriptome

According to the similarity searches of the public databases, 71,746 non-redundant unigenes had at least one annotation result against GO, KEGG, KOG, COG, eggNOG, Swiss-Prot, or Nr databases (Table 1). For the Nr annotation, three BLASTX top-hit species were *Beta vulgaris* subsp. *vulgaris* (2,914, 5.76%), *Chenopodium quinoa* (3,592, 5.29%) and *Spinacia oleracea* (2,369, 3.49%) (Fig. S2). Based on the Blast2GO classified results, 44,293 non-redundant unigenes were assigned to GO terms which usually contain three GO categories: cellular component (CC), molecular function (MF) and biological process (BP). In the CC category, “cell” (21,212) and “cell part” (21,169) were prominent, and catalytic activity (22,308) and binding (20,076) were dominant in the MF category. For the BP category, the greatest number of unigenes were assigned to “metabolic process” (23,029) term followed by “cellular process” (21,582) and “single-organism process” (14,773) (Fig. 1).

**Table 1:** Summary of annotations on unigenes of *R. palmatum* complex against public databases

Databases	Annotated number	300 < = length < 1000	Length > = 1000
COG	25,918	4937	15,788
GO	44,293	10,377	24,943
KEGG	26,727	6,727	15,431
KOG	39,469	8,988	23,579
Pfam	49,027	10,599	30,945
Swissprot	40,179	8843	26,425
eggNOG	65,068	14,833	37,246
Nr	67,942	16,265	38,497
All	71,746	16,990	38,812

The non-redundant unigenes were search against KOG database to get the classification of orthologous proteins, and the results indicated that 39,469 unigenes were assigned into 25 categories (Fig. S3). The three dominant terms were general function prediction only (7867, 17.96%), posttranslational modification, protein turnover, chaperones (4754, 8.81%), and translation, ribosomal structure and biogenesis (3858, 12.07%) (Fig. S3).

In order to predict the candidate pathways and genes related to the specific metabolites, all the non-redundant unigenes were used for the KEGG pathway analyses. Totally, 26,727 annotated genes were assigned to 130 KEGG pathways. Of these pathways, top ten KEGG pathways were ribosome (2,477), carbon metabolism (1,132), biosynthesis of amino acids (1,007), protein processing in endoplasmic reticulum (961), spliceosome (778), oxidative phosphorylation (763), RNA transport (636), endocytosis (603), starch and sucrose metabolism (603) and glycolysis/gluconeogenesis (554) (Fig. S4). The further KEGG enrichment analysis for these candidate pathways indicated that 957 unigenes were enriched in the pathways such as phenylpropanoid biosynthesis (313, 32.71%), terpenoid backbone biosynthesis (158, 16.51%), flavonoid biosynthesis (95, 9.93%), carotenoid biosynthesis (70, 7.31%) etc. (Table S4).

### Quantification of gene expression in top-geoherb and non-geoherb groups

In order to evaluate the gene expression profiles between top-geoherb and non-geoherb groups, FPKM values for each group were calculated and normalized. After calculating the FPKM values, we found that the overall gene expression profiles of top-geoherb and non-geoherb groups showed a different distribution status (Fig. S5). The 28 unigenes with a considerable expression levels (FPKM > 1000) were detected, and 26 of these unigenes were shared by two groups. Functional annotation results indicated that these high expressed genes in two groups were involved in the functions such as stress response, translation, amino acid transport and metabolism, phloem development, cell wall, cytoskeleton, metal ion binding and S-adenosylmethionine biosynthetic process etc. (Table S5). Besides, we noticed that some specific genes showed high expression levels in the non-geoherb samples were annotated in the functions involved in stress response, posttranslational modification, flower development, menaquinone biosynthesis. While the specific genes showed high expression levels in the top-geoherb samples were annotated in the functions related to stress response, nucleotide transport and metabolism, flower development and ion carrier.

**Fig. 2:**
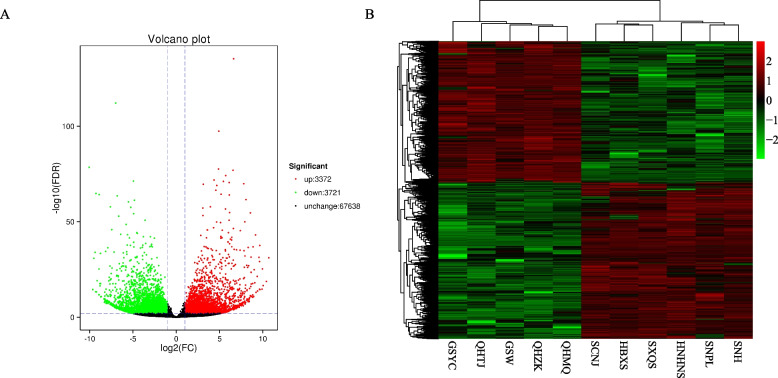
The Volcano plot of differentially expressed genes between top-geoherb and non- geoherb groups

We screened the DEGs between two different groups with DESeq2 analyses. Comparing with the samples derived from the non-geoherb areas, 7,093 differentially expressed unigenes (DEGs) were identified in top-geoherb samples, including 3,372 up-regulated and 3,721 down-regulated unigenes (Fig. 2A). Hierarchical clustering for all the DEGs showed that all samples were clustered into two groups corresponding to the top-geoherb and non-geoherb areas (Fig. 2B). The gene functional prediction results indicated that 5741, 4314, 3480, 3168, 2271, 1978 DEGs could be annotated in NR, Swiss-Prot, GO, KOG, KEGG, COG database, respectively. KEGG enrichment analysis showed that 1,178 DEGs were classified into 122 pathways. These pathways were involved in the plant hormone signal transduction, carotenoid biosynthesis, phenylalanine, tyrosine and tryptophan biosynthesis, cutin, suberine and wax biosynthesis, flavonoid biosynthesis, terpenoid backbone biosynthesis and phenylpropanoid biosynthesis, and the top enriched 24 pathways were presented in Fig. S6. Unexpectedly, no pathways directly related to the biosynthesis of anthraquinones were found in the aforementioned enrichment results, but the highly enriched plant hormone signal transduction pathway related to the interactions of phytohormones which may indirectly promote the accumulation of anthraquinones.

### Genes involved in the biosynthesis of medicinally effective compounds

It has been shown that anthraquinones are the main medicinally effective compounds synthesized by the polyketide pathway and a combination of shikimate and mevalonate (MVA)/methyl-D-erythritol 4-phosphate (MEP) pathways [35, 36]. We therefore screened the abovementioned anthraquinone-related genes. Totally, 68 genes specific to MVA pathway, 23 genes specific to MEP pathway, 62 genes specific to shikimate pathway and 26 genes specific to polyketide pathway were identified in the presented study (Table 2). Of these anthraquinone-related genes, 21 were differentially expressed between top-geoherb and non-geoherb groups (Table S6). As Cyt P450s (CYPs) and UDP-glycosyltransferases (UGTs) may be involved in the oxidation, hydroxylation and glycosylation steps of biosynthetic pathway of anthraquinones. We also screened such genes in the transcriptomes of top-geoherb and non-geoherb samples. We found that 288 genes were predicted to be the members of CYP family, and 118 genes may encode UDP-Glucosyl transferase, and 15 CYP and 14 UGT genes were significantly differentially expressed between comparison groups (Table S7).

**Table 2:** Candidate genes involved in the biosynthesis of anthraquinones of *R. palmatum* complex

Pathway	Gene name	Enzyme symbol	KO number	No
MVA	Acetyl-CoA C-acetyltransferase	*AACT*	K00626	14
	Hydroxymethylglutaryl-CoA synthase	*HMGS*	K01641	10
	Hydroxymethylglutaryl-CoA reductase	*HMGR*	K00021	21
	Mevalonate kinase	*MK*	K00869	4
	Phosphomevalonate kinase	*PMK*	K00938	3
	Diphosphomevalonate decarboxylase	*MPD*	K01597	6
	Isopentenyl-diphosphate Delta-isomerase	*IPPs*	K01823	10
MEP	1-deoxy-D-xylulose-5-phosphate synthase	*DXS*	K01662	9
	1-deoxy-D-xylulose-5-phosphate reductoisomerase	*DXR*	K00099	2
	2-C-Methyl-D-erythritol 4-phosphate cytidylyltransferase	*ISPD*	K00991	1
	4-diphosphocytidyl-2-C-methyl-D-erythritol kinase	*CDPMEK*	K00919	1
	2-C-methyl-D-erythritol 2,4-cyclodiphosphate synthase	*ISPF*	K01770	2
	(E)-4-hydroxy-3-methylbut-2-enyl-diphosphate synthase	*HDS*	K03526	5
Shikimate	4-hydroxy-3-methylbut-2-en-1-yl diphosphate reductase	*HDR*	K03527	3
	3-deoxy-7-phosphoheptulonate synthase	*DAHPS*	K01626	13
	3-dehydroquinate synthase	*DHQS*	K01735	4
	3-dehydroquinate dehydratase / shikimate dehydrogenase	*SDH*	K13832	12
	Shikimate kinase	*SMK*	K00891	8
	3-phosphoshikimate 1-carboxyvinyltransferase	*EPSPs*	K00800	6
	Chorismate synthase	*CS*	K01736	13
	Menaquinone-specific isochorismate synthase	*IS*	K02552	2
	Isochorismate synthase	*MenF*	K14759	0
	2-succinylbenzoate–CoA ligase	*MenE*	K14760	1
	Naphthoate synthase	*MenB*	K01661	3
Polyketide	Type III polyketide synthase	*PKS III*	K00660	26
Glycosylation	UDP-Glucosyl Transferase	*UGT*	_	118
CYP450s	Cytochrome P450	_	_	256
	NADPH-cytochrome P450 reductase	_	_	32

### The correlation between the expression levels of DEGs and the contents of anthraquinones

**Fig. 3:**
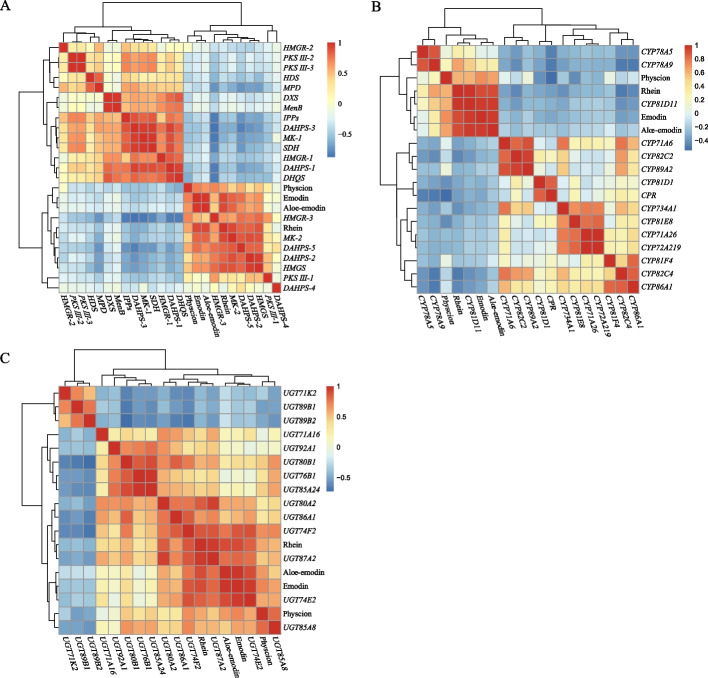
The correlation analyses for the DEGs and contents of five free anthraquinones. **A** The correlations between structural enzyme genes and five free anthraquinones. **B** The correlations between CYPs and five free anthraquinones. **C** The correlations between UGTs and five free anthraquinones

The average contents of five anthraquinones for each sample were recovered from our previous study [28], and the results showed that the average contents of aloe-emodin, rhein, emodin and physcion were higher in top-geoherb group than that in non-geoherb group (Table S8, Fig. S7). Especially, the content of rhein in the top-geoherb group was significantly higher than that in the non-geoherb group (t-test, *P* < 0.05) (Fig. S7). Afterward, the contents of five free anthraquinones were used to infer their correlation with the gene expression of DEGs involved in the biosynthesis of anthraquinones (Fig. 3). The results indicated that five structural genes (*HMGS*, *DAHPS*-2, *DAHPS*-5, *MK*, *HMGR*) in MVA and MEP pathway were positively correlated with the contents of five free anthraquinones, and we also found that *CYP81D11* and 6 *UGT*s (*UGT74E2*, *UGT74F2*, *UGT80A2*, *UGT85A8*, *UGT86A1* and *UGT87A2*) showed significant correlation with five free anthraquinone contents (Fig. 3). Therefore, these aforementioned genes may induce the content difference of anthraquinones for the samples derived from top-geoherb areas and non-geoherb areas and then promote the geoherbalism formation of rhubarb.

### Validation of the DEGs related to the anthraquinone biosynthesis

**Fig. 4:**
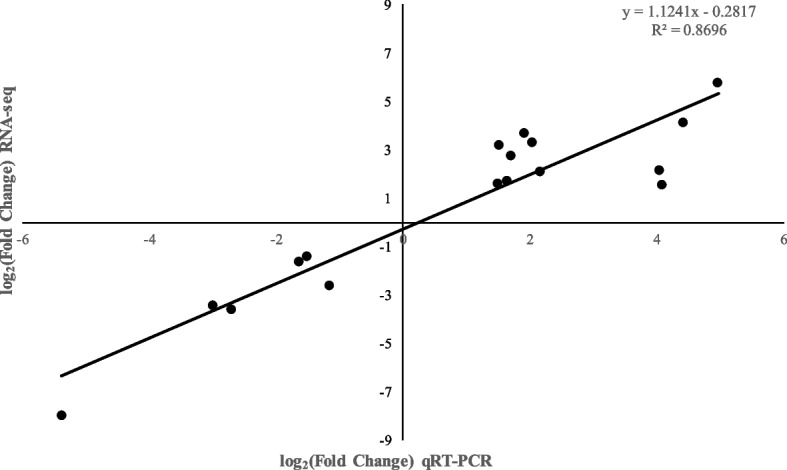
Correlation scatter plot between log_2_^average(2^−ΔΔCT)^ and log_2_^FPKM^, which indicates the relationship between RNA-Seq and qRT-PCR

In order to validate the DEGs detected in the comparison groups in the presented study, 15 candidate DEGs related to anthraquinone biosynthesis were randomly selected for the qRT-PCR. All the dissolution curves of the designed candidate gene primers showed a standard single peak, and no non-specific amplification was produced, indicating that the primers with good specificity could be further used in qRT-PCR experiments (Fig. S8). The results indicated that relative gene expression profiles of qRT-PCR were consistent with the ones from RNA-seq, indicating the accuracy of the identified DEGs and the reliability of transcriptome dataset (Fig. 4).

### Candidate positive-selected genes between top-geoherb and no-geoherb groups

**Fig. 5:**
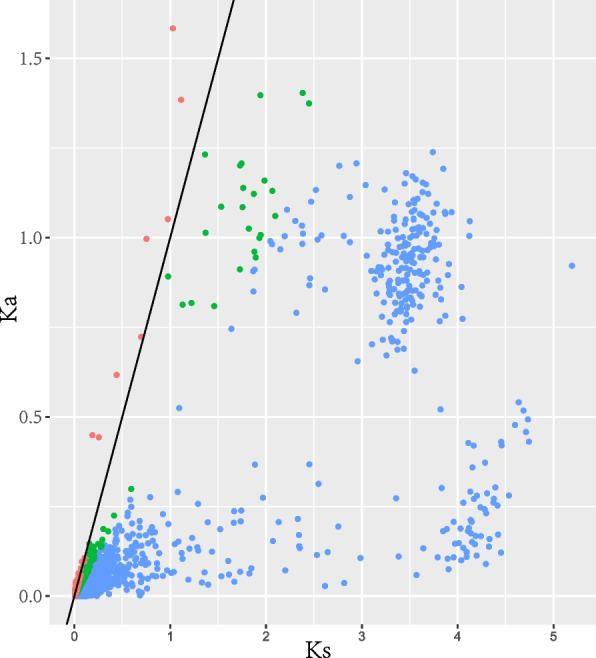
Distribution of Ka and Ks for 7,778 pairs of orthologs between top-geoherb and non-geoherb groups. The dots above the slash indicate the positive-selected genes, and the dots below the slash indicate the negative-selected genes

After ORF prediction, the protein sequences were obtained for two groups and then used to predict the orthologs. Totally, 9,099 candidate single copy nuclear genes (SCNGs) were obtained to estimate substitution rates. 1,321 pairs of these SCNGs only had Ka or Ks values that cannot be used to calculate Ka/Ks values. Finally, 7,778 pairs of SCNGs were retained to calculate Ka, Ks and Ka/Ks values. Of these paired orthologs, 476 pairs had a Ka/Ks value > 1, indicating that these genes have undergone positive selection (Fig. 5). Besides, 1,297 pairs of orthologous genes showed a 1 > Ka/Ks > 0.5, indicating these genes may undergone slight negative selection (Fig. 5). Functional annotations showed that 23 of 476 pairs significant positive-selected genes (*P* < 0.05) were involved in gene expression, substance transport, stress response and metabolism (Table S9).

## Discussion

In past decade, high-throughput transcriptome sequencing has gradually become an economical and efficient way to obtain the comprehensive transcript information as well as provide more insights for the discovery of specific functional genes [37]. Geoherbs are highly appreciated by doctors because of their excellent quality and exact curative effect. One of the important reasons for the formation of geoherbalism might be attributed to the difference in the expression of key enzyme genes involved in specific metabolic pathways among different populations of medicinal plants [4]. Nowadays, more and more researchers have paid attention to the functional genomics of geoherbs and attempt to elucidate the important secondary metabolic pathways and identify the functional enzyme genes related to the biosynthesis of secondary metabolites by using transcriptome sequencing [38].

Especially for non-model medicinal plant species, the paucity of reference genome and the unclear genetic background largely hinder the functional genomic research and the genetic improvement of TCMs. RNA-seq provides a solution for obtaining genome sequences and transcript characterization of non-model plants. In this study, transcriptome sequencing was carried out for the source plants of rhubarb derived from top-geoherb and non-geoherb areas based on Illumina Hiseq X Ten high-throughput sequencing platform, and about 412.32 Gb reads were obtained, providing a large transcriptome dataset for *Rheum* plants than ever [36, 39–41]. The comprehensive transcriptome datasets presented in the current study not only enhanced the genome resources of *Rheum* species, but also laid a solid foundation of research on the growth and development, the biosynthesis of secondary metabolites and the regulation of transcription in source plants of rhubarb.

According to the sequence annotation results, the majority of the non-redundant unigenes (71.30%) showed similarity to the genes from the public databases while the remaining unigenes could not be annotated. We deduced that these unigenes without annotation information may represent novel transcripts or they were matched to untranslated regions. In addition, we found that more than half of the unigenes over 1,000 bp in length had at least one blast hit against in the eight public databases. It has been showed that unigenes with long length were more likely to have BLAST matches in the public databases [17, 42–44]. From the GO annotations, large proportion of unigenes were annotated with the metabolic process, which could be used to identify the new genes related to the secondary metabolic pathways. Besides, a fair number of unigenes were involved in the catalytic activity and binding, indicating that gene regulation and enzyme activity are extremely popular in the roots of *R. palmatum* complex. Previous study also reported that the largest number of genes from the *Coptis chinensis* transcriptome were also enriched in the similar functional categories [45]. We found some candidate genes related to the biosynthesis of phenylpropanoids, terpenoids, flavonoids, carotenoids, alkaloids, anthocyanins. The discovery of these genes lays a foundation for analyzing the biosynthesis pathway of important secondary metabolites of rhubarb, and then provides a theoretical basis for revealing the biosynthesis mechanism of specific secondary metabolites.

Rhubarb has a wide range of pharmacological activities due to its high content of anthraquinones which may be synthesized by a polyketide pathway and a combination of shikimate and mevalonate/methyl-D-erythritol 4-phosphate pathways [35, 46–48]. The study on the biosynthesis and transcriptional regulation mechanism of anthraquinones will help to artificially manipulate its secondary metabolic pathway and reconstruct the biosynthetic pathway with bioengineering to enhance the yield of anthraquinones. Previously, we identified the candidate enzyme genes involved in the MVA, MEP, shikimate and polyketide pathways for *R. tanguticum* and *R. officinale*, which provide insights for the inference of the accumulation differences of anthraquinones in different tissues for *Rheum* species [36, 49]. In the present study, anthraquinone-related enzyme genes were also identified, and these genes will provide more candidates for the genetic manipulation of anthraquinone biosynthesis in *R. palmatum* complex. In addition, the expression profiles of anthraquinone-related genes were found to be correlated with the contents of free anthraquinones in the *R. palmatum* complex derived from different areas, which further confirmed that the geoherbalism formation of medicinal plants is partly attributed to the difference in the expression of key enzyme genes related to the biosynthesis pathway of active components under different environments [4].

It has been shown that the chemical composition for medicinal materials derived from top-geoherb areas is the result of their adaptation to the special habitat, indicating that adaptiveness of medicinal plants to complicated systems [4]. As a representative geoherb, the geoherbalism of rhubarb might be closely related to the adaptive evolution of its source plants under the natural selection. Here, we estimated the Ka/Ks ratios for the paired orthologs shared by top-geoherb and non-geoherb groups to investigate the effects of the natural selection pressures on the rhubarb. Our results indicated that most genes have undergone negative selection and that a low proportion of orthologous genes were subjected to positive selection. Therefore, we inferred that the geoherbalism may be affected by the natural selection. In addition, previous study mentioned that the features of geoherbalism are developed through the microevolution of quantitative genetics controlled by multiple genes under environmental stress [4]. We noticed that some of positive-selected genes were related to the stress response and metabolism, which further confirmed that the specific stress induced by the environmental heterogeneity might have a profound effect on the characters of geoherbalism for rhubarb.

## Conclusion

In the present study, comprehensive transcriptome datasets were obtained for *R. palmatum* complex derived from different areas. The large numbers of unigene datasets obtained in this study not only enhanced the genomic resources for *Rheum* species, but also provided a robust genetic basis for the identification of key genes related to the biosynthesis of medicinally effective compounds in *R. palmatum* complex. The comparative transcriptome analyses showed that some DEGs detected between top-geoherb and non-geoherb groups of *R. palmatum* complex were involved in the biosynthesis of anthraquinone, indicating that geoherbalism formation for rhubarb was influenced by the specific gene expression profiles. Besides, there was evidence that natural selection altered the protein structures of environment-related genes affecting the geoherbalism for *R. palmatum* complex which are growing in different environments. These findings provide insight into the molecular mechanism of geoherbalism for rhubarb and are important cues for the quality improvement of rhubarb germplasms.

### Supplementary Information

**Supplementary Material 1.**

**Supplementary Material 2.**

**Supplementary Material 3.**

**Supplementary Material 4.**

**Supplementary Material 5.**

**Supplementary Material 6.**

**Supplementary Material 7.**

**Supplementary Material 8.**

**Supplementary Material 9.**

## Data Availability

All the raw reads generated in this study have been deposited in the NCBI with the BioProject accession number PRJNA961302 (https://www.ncbi.nlm.nih.gov/bioproject/PRJNA961302).
