# Supplementary material for: Transcriptomic divergence of the Rheum palmatum complex derived from top-geoherb and non-geoherb areas provides the insights into geoherbalism properties of rhubarb
Source: BMC Genomics. 2024 Feb 26;25:212. doi: 10.1186/s12864-024-10142-3 (PMC10898026; doi:10.1186/s12864-024-10142-3)
Supplement: Supplementary file 1 — Supplementary Material 1. [file 12864_2024_10142_MOESM1_ESM.docx]

**Table S1** Information of 11sample sites for *R. palmatum* complex

| Region | Location | ID | Species | Voucher IDs | Altitude (m) | Latitude | Longitude | No. |
| --- | --- | --- | --- | --- | --- | --- | --- | --- |
| Top-geoherb area | Wenxian, Gansu | GSW | *R. palmatum* | GSW201901-GSW201905 | 3285 | 32°50' | 104°24' | 5 |
|  | Yongchang, Gansu | GSYC | *R. palmatum* | GSYC201901-GSYC201905 | 3006 | 38°06' | 101°51' | 5 |
|  | Maqin, Qinghai | QHMQ | *R. tanguticum* | QHMQ201901-QHMQ201905 | 3391 | 34°39' | 100°37' | 5 |
|  | Tianjun, Qinghai | QHTJ | *R. tanguticum* | QHTJ201901-QHTJ201905 | 3774 | 37°06' | 98°56' | 5 |
|  | Zeku, Qinghai | QHZK | *R. tanguticum* | QHZK201901-QHZK201905 | 3481 | 35°15' | 101°53' | 5 |
| Non-geoherb area | Xinshan, Hubei | HBXS | *R. officinale* | HBXS201901-HBXS201905 | 2870 | 31°26' | 110°15' | 5 |
|  | Neixiang, Henan | HNHNS | *R. officinale* | HNHNS201901-HNHNS201905 | 1463 | 33°28' | 111°55' | 5 |
|  | Huxian, Shaanxi | SNH | *R. palmatum* | SNH201901-SNH201905 | 2239 | 33°50' | 108°47' | 5 |
|  | Pingli, Shaaxi | SNPL | *R. officinale* | SNPL201901-SNPL201905 | 2884 | 32°01' | 109°21' | 5 |
|  | Qinshui, Shanxi | SXQS | *R. palmatum* | SXQS201901-SXQS201905 | 2267 | 35°25' | 111°57' | 5 |
|  | Nanjiang, Sichuan | SCNJ | *R. officinale* | SCNJ201901-SCNJ201905 | 1822 | 32°35' | 107°06' | 5 |

**Table S2** Primer pairs for the qRT-PCR validation.

| Primer name | Forward primer (5′–3′) | Reverse primer (5′–3′) |
| --- | --- | --- |
| *CYP72A219* | CGCCAAGTACAACGAGTTCCAGAA | CCACAAGCTCACCATCTCACTACAG |
| *SDH* | CCGTGGAACTTGGTGCTAACTACAT | ACAAGACTGCCAATCTCCTCAACTG |
| *UGT89B2* | TGACGGCGGACTACCTGTATGATT | CCTCCATTCCTTCCTCTTCCTCCAT |
| *NADPH* | CATACTGATAAGGAGGACGGCACAC | TAACGCAAGCAAGGCAGACTTCTT |
| *CYP81D1* | TCCTAACACCACACGAATCCTCAGA | TTACCCTTACCACCGCCCTCAAA |
| *IPPs* | CCAGATGAGGTTGCTGATGTCAAGT | CCACAAGCCTGAACCAAGGAGAC |
| *CYP86A1* | GCTGGTTCTTCTGGCTCGTCAT | AAGGTCAACGGCTCCTCTATCCA |
| *MenB* | TGTCACGCATCGGAGGTCACTT | CGCTGGAATCTGTCCTTGGTTGAG |
| *CYP82C4* | TCATCACACCGCCGACACCAA | AGCAATGACACGAGCCAAGTTAGG |
| *MPD* | GCTGGGTTGGCATCTTCTGTTG | CTTCATCATCAGCACCAGCACCA |
| *HMGS* | GCGTCTGGAAGTTGGCAGTGAA | CAAAGCAGCAGTTCCTCCATAGCA |
| *PKS Ⅲ-1* | ACGGTGCTGCGGCTGTCATA | GGGAACGGTCTGGTAGAGTTGGAA |
| *UGT74F2* | GCTTGAGCCTCAGGTGGTGGAT | GGTGGTTGTGGAGTGGTTGAAGTT |
| *HMGR-3* | GCAAACATGACAACGGGCAACAA | GAATGGCGGTGGAGGAAGCATAG |
| *DAHPS-5* | GAACTCGCTCACCGTGTTGATGA | TCTAGTCAGTGCCTGCTCGTATGG |

**Table S3** Sequencing information of all samples

| Sample ID | Number of reads | Base number | GC (%) | Q20 (%) | Q30 (%) |
| --- | --- | --- | --- | --- | --- |
| HBXS01 | 23,148,002 | 6,869,619,438 | 48.68 | 96.91 | 91.98 |
| HBXS02 | 26,736,584 | 7,976,305,064 | 48.32 | 97.04 | 92.22 |
| HBXS03 | 28,740,941 | 8,578,321,922 | 48.50 | 96.80 | 91.78 |
| HBXS04 | 24,121,884 | 7,178,671,820 | 49.10 | 96.85 | 91.91 |
| HBXS05 | 31,551,475 | 9,388,256,838 | 48.50 | 96.99 | 92.18 |
| HNHNS01 | 25,067,178 | 7,465,012,434 | 47.81 | 96.93 | 92.00 |
| HNHNS02 | 24,560,107 | 7,285,527,602 | 48.05 | 97.37 | 92.91 |
| HNHNS03 | 25,287,340 | 7,520,159,334 | 47.98 | 97.06 | 92.23 |
| HNHNS04 | 25,845,665 | 7,687,589,526 | 47.94 | 97.31 | 92.79 |
| HNHNS05 | 26,318,370 | 7,827,444,308 | 47.98 | 97.02 | 92.17 |
| SCNJ01 | 34,946,866 | 10,401,308,762 | 48.29 | 97.02 | 92.18 |
| SCNJ02 | 25,734,227 | 7,678,517,760 | 48.37 | 96.91 | 92.08 |
| SCNJ03 | 27,552,685 | 8,196,064,848 | 47.92 | 97.23 | 92.65 |
| SCNJ04 | 27,277,646 | 8,131,837,206 | 48.08 | 97.05 | 92.30 |
| SCNJ05 | 26,408,596 | 7,893,096,190 | 49.61 | 96.86 | 91.98 |
| SNH01 | 21,436,968 | 6,375,237,052 | 48.40 | 97.17 | 92.48 |
| SNH02 | 24,946,869 | 7,411,760,670 | 48.44 | 97.33 | 92.82 |
| SNH03 | 24,658,324 | 7,325,951,288 | 49.42 | 97.21 | 92.56 |
| SNH04 | 22,726,885 | 6,777,751,742 | 49.01 | 97.12 | 92.55 |
| SNH05 | 21,938,146 | 6,549,442,982 | 48.46 | 96.88 | 92.00 |
| SNPL01 | 28,733,563 | 8,537,669,624 | 48.98 | 97.44 | 93.01 |
| SNPL02 | 22,654,367 | 6,735,892,360 | 48.41 | 97.17 | 92.50 |
| SNPL03 | 22,160,622 | 6,598,807,598 | 48.64 | 96.98 | 92.08 |
| SNPL04 | 22,162,453 | 6,603,777,658 | 48.67 | 97.01 | 92.10 |
| SNPL05 | 25,059,059 | 7,441,450,556 | 48.27 | 97.22 | 92.59 |
| SXQS01 | 30,310,981 | 9,058,784,676 | 48.79 | 96.70 | 91.67 |
| SXQS02 | 23,415,205 | 6,969,972,828 | 49.06 | 97.04 | 92.31 |
| SXQS03 | 28,007,601 | 8,366,783,802 | 48.58 | 96.86 | 91.92 |
| SXQS04 | 24,678,345 | 7,334,229,778 | 48.93 | 96.58 | 91.26 |
| SXQS05 | 25,326,001 | 7,550,767,606 | 48.01 | 96.91 | 92.00 |
| GSW01 | 25,100,307 | 7,493,353,830 | 48.89 | 96.88 | 91.95 |
| GSW02 | 25,079,186 | 7,496,033,188 | 49.24 | 96.92 | 92.11 |
| GSW03 | 21,867,970 | 6,523,348,700 | 48.59 | 96.73 | 91.67 |
| GSW04 | 28,413,527 | 8,480,928,058 | 48.54 | 97.03 | 92.28 |
| GSW05 | 26,808,850 | 7,995,481,332 | 48.54 | 96.96 | 92.09 |
| GSYC01 | 23,600,564 | 7,031,646,614 | 48.02 | 96.91 | 92.03 |
| GSYC02 | 23,350,031 | 6,943,563,074 | 48.23 | 96.92 | 92.01 |
| GSYC03 | 21,833,626 | 6,499,172,682 | 50.56 | 96.77 | 91.89 |
| GSYC04 | 26,027,623 | 7,754,459,072 | 48.09 | 96.86 | 91.96 |
| GSYC05 | 28,199,277 | 8,397,430,106 | 48.25 | 96.97 | 92.14 |
| QHMQ01 | 21,819,047 | 6,498,602,316 | 48.74 | 97.00 | 92.18 |
| QHMQ02 | 26,372,101 | 7,833,345,498 | 48.11 | 97.06 | 92.24 |
| QHMQ03 | 24,115,877 | 7,169,490,602 | 49.23 | 97.16 | 92.48 |
| QHMQ04 | 24,718,382 | 7,349,857,110 | 48.64 | 96.92 | 91.94 |
| QHMQ05 | 22,603,494 | 6,736,029,152 | 48.52 | 96.54 | 91.29 |
| QHTJ01 | 25,476,531 | 7,588,160,542 | 48.01 | 96.97 | 92.09 |
| QHTJ02 | 25,226,078 | 7,518,828,756 | 48.56 | 97.18 | 92.44 |
| QHTJ03 | 22,881,908 | 6,808,012,694 | 48.31 | 96.96 | 92.02 |
| QHTJ04 | 22,885,666 | 6,807,288,448 | 48.81 | 97.15 | 92.44 |
| QHTJ05 | 25,940,548 | 7,703,020,314 | 48.64 | 97.12 | 92.41 |
| QHZK01 | 25,989,435 | 7,737,657,338 | 48.81 | 96.64 | 91.40 |
| QHZK02 | 22,810,905 | 6,788,003,620 | 48.36 | 96.92 | 92.01 |
| QHZK03 | 23,629,281 | 7,023,638,288 | 48.16 | 97.12 | 92.39 |
| QHZK04 | 26,980,620 | 8,029,152,214 | 48.07 | 97.12 | 92.36 |
| QHZK05 | 21,472,983 | 6,392,504,064 | 48.13 | 97.08 | 92.34 |

**Table S4** KEGG pathway enrichment of unigenes for *R. palmatum* complex

| Pathway | Unigene No. | Percent | ko_ ID |
| --- | --- | --- | --- |
| Phenylpropanoid biosynthesis | 313 | 32.71% | ko00940 |
| Terpenoid backbone biosynthesis | 158 | 16.51% | ko00900 |
| Flavonoid biosynthesis | 95 | 9.93% | ko00941 |
| Carotenoid biosynthesis | 70 | 7.31% | ko00906 |
| Isoquinoline alkaloid biosynthesis | 69 | 7.21% | ko00950 |
| Tropane, piperidine and pyridine alkaloid biosynthesis | 57 | 5.96% | ko00960 |
| Stilbenoid, diarylheptanoid and gingerol biosynthesis | 39 | 4.08% | ko00945 |
| Sesquiterpenoid and triterpenoid biosynthesis | 32 | 3.34% | ko00909 |
| Zeatin biosynthesis | 26 | 2.72% | ko00908 |
| Diterpenoid biosynthesis | 22 | 2.30% | ko00904 |
| Caffeine metabolism | 18 | 1.88% | ko00232 |
| Brassinosteroid biosynthesis | 16 | 1.67% | ko00905 |
| Limonene and pinene degradation | 13 | 1.36% | ko00903 |
| Betalain biosynthesis | 9 | 0.94% | ko00965 |
| Monoterpenoid biosynthesis | 8 | 0.84% | ko00902 |
| Flavone and flavonol biosynthesis | 7 | 0.73% | ko00944 |
| Anthocyanin biosynthesis | 3 | 0.31% | ko00942 |
| Glucosinolate biosynthesis | 2 | 0.21% | ko00966 |

**Table S5** The highly expressed genes in top-geoberb and non-geoherb groups of *R. palmatum* complex

| ID | Length | FPKM | | Gene name | Predicted function |
| --- | --- | --- | --- | --- | --- |
|  | （bp） | Top-geoherb area | Non-geoherb area |  |  |
| Unigene_0003299 | 2,088 | 2036.56 | 2846.19 | S-adenosylhomocysteine hydrolase | S-adenosylmethionine biosynthetic process |
| Unigene_0006146 | 1,155 | 1397.54 | 1086.94 | polyubiquitin 11-like | Stress response |
| Unigene_0027715 | 1,394 | 1231.89 | 1173.80 | Glycine-rich RNA-binding protein | Translation, ribosomal structure and biogenesis |
| Unigene_0028505 | 6,541 | 4306.82 | 6332.47 | hypothetical protein GLYMA_13G021700 | General function prediction |
| Unigene_0032526 | 5,245 | 1125.01 | 1473.83 | Leucine aminopeptidase 2, chloroplastic | Amino acid transport and metabolism |
| Unigene_0225938 | 1,701 | 2866.22 | 1883.98 | DnaJ protein homolog | Stress response |
| Unigene_0236109 | 567 | 11260.71 | 4405.70 | Metallothionein-like protein type 2 | Metal ion binding |
| Unigene_0238870 | 916 | 3997.70 | 1570.02 | MLP-like protein 31 | Stress response |
| Unigene_0241079 | 990 | 3708.17 | 2316.74 | Defensin J1-2 | Defense response |
| Unigene_0243297 | 460 | 1075.19 | 1462.33 | HSP70 | Stress response |
| Unigene_0247752 | 2,482 | 3284.66 | 3552.75 | HSP70 | Stress response |
| Unigene_0248755 | 914 | 1440.18 | 5663.70 | Phloem protein 2 | Phloem development |
| Unigene_0251261 | 867 | 1262.08 | 1433.35 | Xyloglucan endotransglucosylase/hydrolase protein 22 | Cell wall |
| Unigene_0252064 | 2,171 | 2225.68 | 2351.78 | Translation elongation factor 1 | Translation, ribosomal structure and biogenesis |
| Unigene_0252462 | 306 | 1010.06 | 1142.01 | Actin-1 | Cytoskeleton |
| Unigene_0253928 | 762 | 1173.55 | 1092.67 | 40S ribosomal protein S25-2 | Translation, ribosomal structure and biogenesis |
| Unigene_0256212 | 695 | 3746.18 | 1864.29 | Auxin-repressed 12.5 kDa protein | Plant growth |
| Unigene_0256381 | 673 | 1401.66 | 1413.89 | DnaJ protein homolog 2 | Stress response |
| Unigene_0883031 | 491 | 1826.47 | 1799.07 | S-adenosylmethionine synthase 1 | S-adenosylmethionine biosynthetic process |
| Unigene_0885692 | 491 | 1406.53 | 1135.07 | Polyubiquitin | Stress response |
| Unigene_0885913 | 1674 | 1624.52 | 1614.46 | Translationally-controlled tumor protein homolog | Translation, ribosomal structure and biogenesis |
| Unigene_0889176 | 1,078 | 1051.58 | 1150.39 | Translation elongation factor 1 | Translation, ribosomal structure and biogenesis |
| Unigene_0889178 | 1,194 | 1326.05 | 2102.42 | Translation elongation factor 1 | Translation, ribosomal structure and biogenesis |
| Unigene_1231524 | 731 | 1003.62 | 1024.19 | Polyadenylate-binding protein RBP45 | Translation, ribosomal structure and biogenesis |
| Unigene_1239240 | 450 | 1356.84 | 1517.20 | Abscisic stress-ripening protein 2 | fruit development |
| Unigene_1246716 | 794 | 1701.29 | 1621.87 | Phloem protein 2 | Phloem development |

**Table S6** DEGs related to the biosynthesis of anthraquinones between top-geoberb and non-geoherb groups of *R. palmatum* complex.

| Gene name | ID | Length | FPKM | | FDR |
| --- | --- | --- | --- | --- | --- |
|  |  | (bp) | Non-geoherb group | top-geoherb group |  |
| *PKS Ⅲ-1* | Unigene_0887843 | 1763 | 0.17 | 80.58 | 1.38E-14 |
| *PKS Ⅲ-2* | Unigene_1230604 | 264 | 32.55 | 6.67 | 6.06E-03 |
| *PKS Ⅲ-3* | Unigene_1231630 | 361 | 41.34 | 9.18 | 1.73E-03 |
| *DAHPS-1* | Unigene_0022649 | 972 | 4.43 | 0.33 | 1.10E-10 |
| *DAHPS-2* | Unigene_0047493 | 1402 | 2.27 | 9.86 | 2.97E-04 |
| *DAHPS-3* | Unigene_0243126 | 3341 | 63.49 | 5.98 | 2.57E-12 |
| *DAHPS-4* | Unigene_1219440 | 1820 | 0.04 | 0.74 | 9.52E-03 |
| *DAHPS-5* | Unigene_1244885 | 3268 | 7.82 | 83.84 | 1.05E-07 |
| *DHQS* | Unigene_0030497 | 3775 | 4.99 | 1.55 | 1.49E-03 |
| *DXS* | Unigene_0254372 | 1710 | 48.19 | 2.14 | 4.11E-04 |
| *HDS* | Unigene_0252768 | 1851 | 4.07 | 0.29 | 3.25E-04 |
| *HMGR-1* | Unigene_0233775 | 1189 | 3.75 | 0.33 | 2.19E-05 |
| *HMGR-2* | Unigene_0245543 | 1521 | 2.60 | 0.36 | 6.08E-03 |
| *HMGR-3* | Unigene_0893618 | 3494 | 0.72 | 4.27 | 7.76E-06 |
| *HMGS* | Unigene_0886732 | 2743 | 1.84 | 4.90 | 7.56E-04 |
| *IPPs* | Unigene_0234626 | 1943 | 4.02 | 0.40 | 6.05E-10 |
| *MenB* | Unigene_0246450 | 1554 | 535.11 | 32.44 | 1.25E-03 |
| *MK-1* | Unigene_0251783 | 5157 | 3.93 | 1.19 | 1.23E-04 |
| *MK-2* | Unigene_0882887 | 3872 | 3.11 | 9.37 | 6.61E-03 |
| *MPD* | Unigene_0255497 | 1539 | 10.36 | 0.19 | 3.66E-19 |
| *SDH* | Unigene_0009293 | 3118 | 10.44 | 3.11 | 7.77E-07 |

**Table S7** Differentially expressed CYPs and UGTs between top-geoberb and non-geoherb groups of *R. palmatum* complex.

| Gene name | ID | length | FPKM | | FDR |
| --- | --- | --- | --- | --- | --- |
|  |  | (bp) | Non-geoherb group | top-geoherb group |  |
| NADPH-cytochrome p450 reductase | Unigene_0042642 | 3,691 | 78.39 | 25.25 | 3.73E-03 |
| Cytochrome P450 71A26 | Unigene_0036222 | 1,340 | 2.55 | 0.01 | 9.76E-03 |
| Cytochrome P450 71A6 | Unigene_0012800 | 1,965 | 1.51 | 0.02 | 2.45E-05 |
| Cytochrome P450 72A219 | Unigene_0001984 | 1,891 | 143.33 | 13.00 | 5.06E-04 |
| Cytochrome P450 734A1 | Unigene_0244657 | 2,250 | 1.22 | 0.08 | 3.82E-04 |
| Cytochrome P450 78A5 | Unigene_0882839 | 2,422 | 0.39 | 12.55 | 2.54E-06 |
| Cytochrome P450 78A9 | Unigene_0028900 | 1,784 | 0.27 | 2.66 | 6.20E-04 |
| Cytochrome P450 81D1 | Unigene_0220186 | 1,864 | 48.66 | 2.73 | 6.03E-07 |
| Cytochrome P450 81D11 | Unigene_1243959 | 2,717 | 0.62 | 72.65 | 1.92E-08 |
| Cytochrome P450 81E8 | Unigene_1231020 | 1,077 | 2.28 | 0.13 | 5.89E-03 |
| Cytochrome P450 81F4 | Unigene_0211309 | 1,773 | 0.63 | 0.00 | 7.16E-03 |
| Cytochrome P450 82C2 | Unigene_0255477 | 1,957 | 9.21 | 1.35 | 1.21E-03 |
| Cytochrome P450 82C4 | Unigene_0255224 | 5,766 | 5.83 | 1.35 | 1.38E-05 |
| Cytochrome P450 86A1 | Unigene_0243037 | 4,109 | 6.50 | 0.71 | 5.02E-06 |
| Cytochrome P450 89A2 | Unigene_0244567 | 2,650 | 4.23 | 0.44 | 4.08E-05 |
| UDP-Glycosyltransferase 71A16 | Unigene_1245968 | 661 | 0.03 | 1.38 | 3.89E-03 |
| UDP -Glycosyltransferase 71K2 | Unigene_0247865 | 2,885 | 27.89 | 1.31 | 3.70E-16 |
| UDP -Glycosyltransferase 74E2 | Unigene_0889352 | 1,833 | 0.33 | 4.59 | 1.14E-03 |
| UDP-Glycosyltransferase 74F2 | Unigene_0891121 | 2,126 | 1.00 | 12.04 | 1.41E-03 |
| UDP-Glycosyltransferase 76B1 | Unigene_0032857 | 4,547 | 0.02 | 1.40 | 6.33E-07 |
| UDP-Glycosyltransferase 80A2 | Unigene_0041579 | 5,002 | 0.55 | 2.03 | 7.65E-05 |
| UDP-Glycosyltransferase 80B1 | Unigene_1249568 | 3,464 | 0.20 | 6.08 | 1.10E-09 |
| UDP-Glycosyltransferase 85A24 | Unigene_0884582 | 2,536 | 0.33 | 3.92 | 1.69E-06 |
| UDP-Glycosyltransferase 85A8 | Unigene_1241209 | 1,225 | 0.00 | 1.72 | 1.02E-06 |
| UDP-Glycosyltransferase 86A1 | Unigene_0888306 | 1,148 | 0.19 | 5.31 | 8.33E-13 |
| UDP-Glycosyltransferase 87A2 | Unigene_0026258 | 1,788 | 2.97 | 10.62 | 3.72E-05 |
| UDP-Glycosyltransferase 89B1 | Unigene_0205447 | 645 | 3.01 | 0.38 | 3.49E-03 |
| UDP-Glycosyltransferase 89B2 | Unigene_0040976 | 1,818 | 26.67 | 2.04 | 1.16E-05 |
| UDP-Glycosyltransferase 92A1 | Unigene_0886900 | 2,394 | 0.33 | 6.92 | 3.19E-04 |

**Table S8** Determination of free anthraquinones contents in 11 populations of *R. palmatum* complex (n=3, mg/g)

| **Region** | **ID** | **rhein** | **emodin** | **chrysophanol** | **aloe-emodin** | **physcion** |
| --- | --- | --- | --- | --- | --- | --- |
| Top-geoberb | GSW | 0.80 | 0.60 | 1.90 | 1.00 | 0.50 |
|  | GSYC | 1.40 | 0.70 | 1.20 | 0.90 | 1.60 |
|  | QHMQ | 3.60 | 0.40 | 1.50 | 1.50 | 0.30 |
|  | QHTJ | 8.70 | 4.30 | 8.60 | 9.00 | 1.80 |
|  | QHZK | 4.30 | 0.90 | 4.00 | 2.50 | 0.90 |
| Non-geoberb | HBXS | 0.90 | 0.30 | 3.30 | 2.20 | 1.00 |
|  | HNHNX | 0.70 | 0.10 | 1.60 | 0.90 | 0.30 |
|  | SNH | 0.70 | 0.10 | 1.50 | 1.60 | 0.20 |
|  | SNPL | 0.60 | 0.20 | 0.70 | 0.70 | 0.20 |
|  | SXQS | 0.30 | 0.10 | 0.70 | 1.00 | 0.20 |
|  | SCNJ | 0.80 | 0.50 | 6.00 | 1.40 | 1.00 |

**Table S9** Functional annotations of 23 pairs significant positive-selected genes between top-geoberb and non-geoherb groups of *R. palmatum* complex.

| Gene ID | | Ka/Ks | *P* value (Fisher) | Predicted function |
| --- | --- | --- | --- | --- |
| Non-geoherb group | Top-geoherb group |  |  |  |
| NG_Unigene_312697 | TG_Unigene_108139 | 2.16 | 0.0001 | Retrovirus-related Pol polyprotein from transposon RE1 |
| NG_Unigene_251591 | TG_Unigene_121283 | 1.52 | 0.0441 | Catechol 1,2-dioxygenase |
| NG_Unigene_263576 | TG_Unigene_89275 | 4.07 | 0.0438 | Hypothetical protein ARMSODRAFT_1012230 |
| NG_Unigene_269291 | TG_Unigene_74589 | 3.43 | 0.0000 | Heptahelical transmembrane protein 1 |
| NG_Unigene_273626 | TG_Unigene_77963 | 7.41 | 0.0221 | Hypothetical protein ARMSODRAFT_1012429 |
| NG_Unigene_277091 | TG_Unigene_45233 | 2.16 | 0.0001 | Pyruvate kinase |
| NG_Unigene_279782 | TG_Unigene_78700 | 3.68 | 0.0241 | Cation/H(+) antiporter 15-like |
| NG_Unigene_282033 | TG_Unigene_94051 | 7.24 | 0.0128 | Putative disease resistance protein At1g50180 |
| NG_Unigene_289062 | TG_Unigene_92265 | 8.72 | 0.0081 | Hypothetical protein ARMSODRAFT_953687 |
| NG_Unigene_290157 | TG_Unigene_89129 | 1.43 | 0.0257 | Sugar transport protein MST3 |
| NG_Unigene_291560 | TG_Unigene_105979 | 4.82 | 0.0307 | 60S ribosomal protein L12-3 |
| NG_Unigene_297082 | TG_Unigene_60504 | 2.62 | 0.0000 | Hypothetical protein RSOLAG1IB_08161 |
| NG_Unigene_297928 | TG_Unigene_83173 | 5.76 | 0.0399 | Aerolysin-like protein FEM32 |
| NG_Unigene_310780 | TG_Unigene_93976 | 9.67 | 0.0359 | Pentatricopeptide repeat-containing protein |
| NG_Unigene_315592 | TG_Unigene_115431 | 2.18 | 0.0340 | Protein of unknown function (DUF1668) |
| NG_Unigene_315629 | TG_Unigene_105133 | 2.49 | 0.0045 | Leucine-rich repeat receptor-like serine/threonine-protein kinase |
| NG_Unigene_316619 | TG_Unigene_112353 | 1.81 | 0.0321 | Protein of unknown function (DUF1668) |
| NG_Unigene_317352 | TG_Unigene_117321 | 8.75 | 0.0001 | Cytochrome P450 71A1 |
| NG_Unigene_326003 | TG_Unigene_103912 | 1.95 | 0.0343 | Uncharacterized protein LOC8278885 |
| NG_Unigene_409425 | TG_Unigene_100563 | 3.28 | 0.0076 | Putative 12-oxophytodienoate reductase 6 |
| NG_Unigene_212532 | TG_Unigene_75324 | 2.14 | 0.0003 | Myo-inositol-1-phosphate synthase |
| NG_Unigene_277183 | TG_Unigene_206380 | 1.94 | 0.0004 | Hypothetical protein G647_08967 |
| NG_Unigene_298542 | TG_Unigene_79218 | 1.38 | 0.0467 | Uncharacterized mitochondrial protein AtMg00810-like |
