# Supplementary figures and images for: Transcriptomic divergence of the Rheum palmatum complex derived from top-geoherb and non-geoherb areas provides the insights into geoherbalism properties of rhubarb
Source: BMC Genomics. 2024 Feb 26;25:212. doi: 10.1186/s12864-024-10142-3 (PMC10898026; doi:10.1186/s12864-024-10142-3)

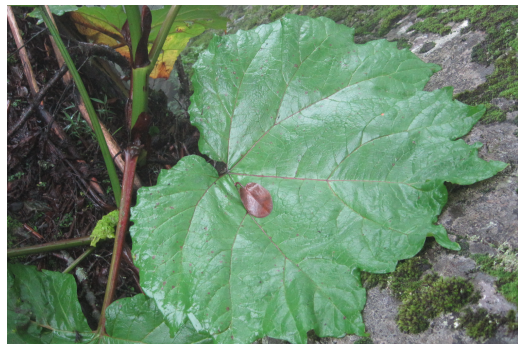

HBXS

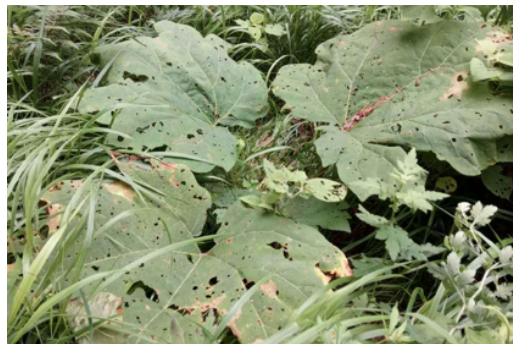

HNHNS

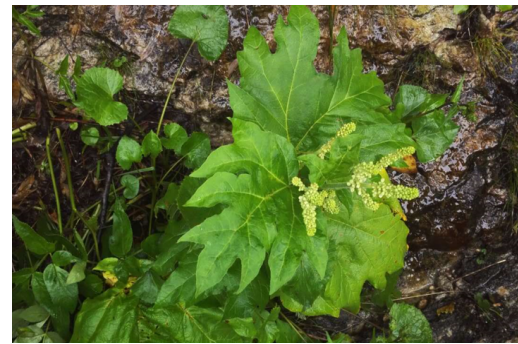

SNH

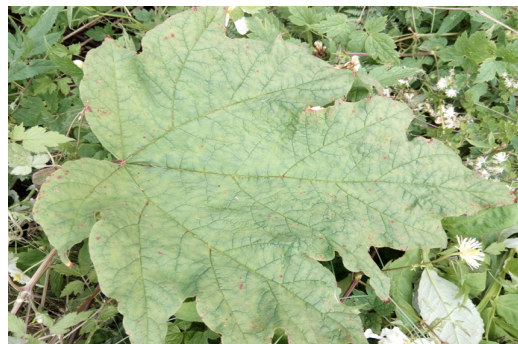

SNPL

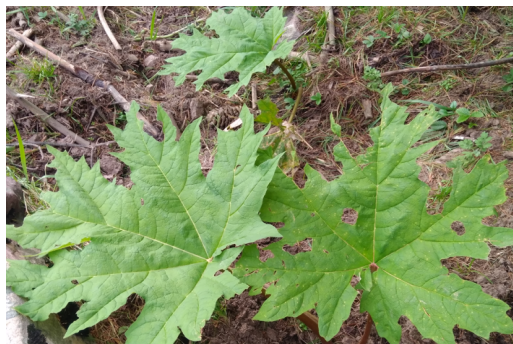

SXQS

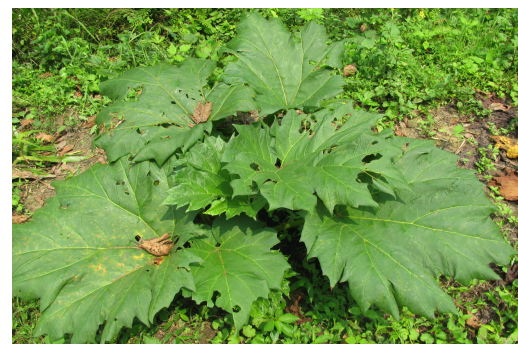

SCNJ

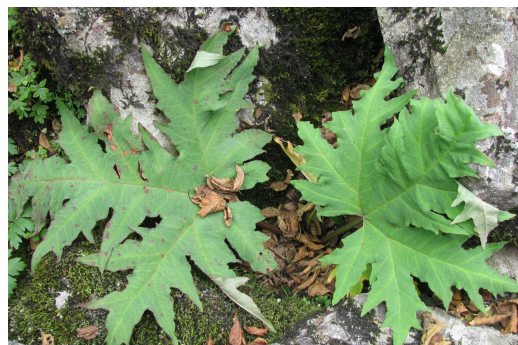

GSW

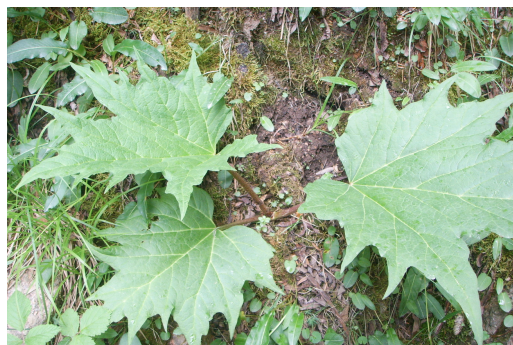

GSYC

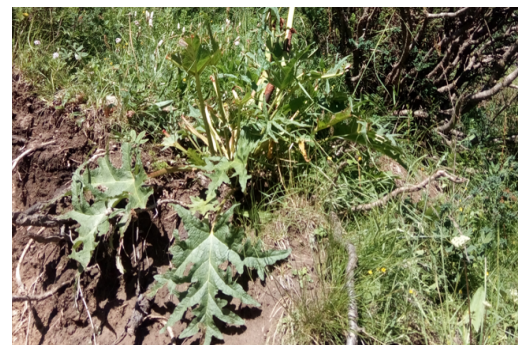

QHMQ

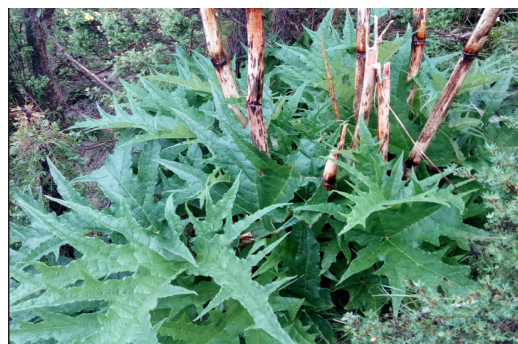

QHTJ

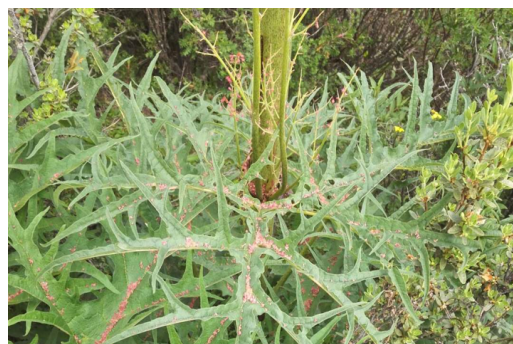

QHZK

Supplement: Supplementary file 2 — Supplementary Material 2. [file 12864_2024_10142_MOESM2_ESM.pdf]

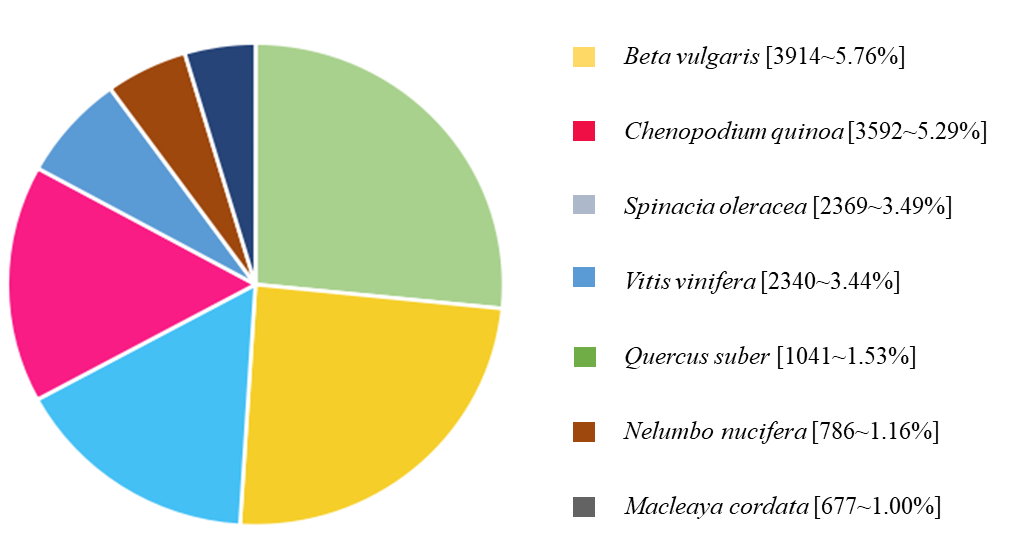

Supplement: Supplementary file 3 — Supplementary Material 3. [file 12864_2024_10142_MOESM3_ESM.tif]

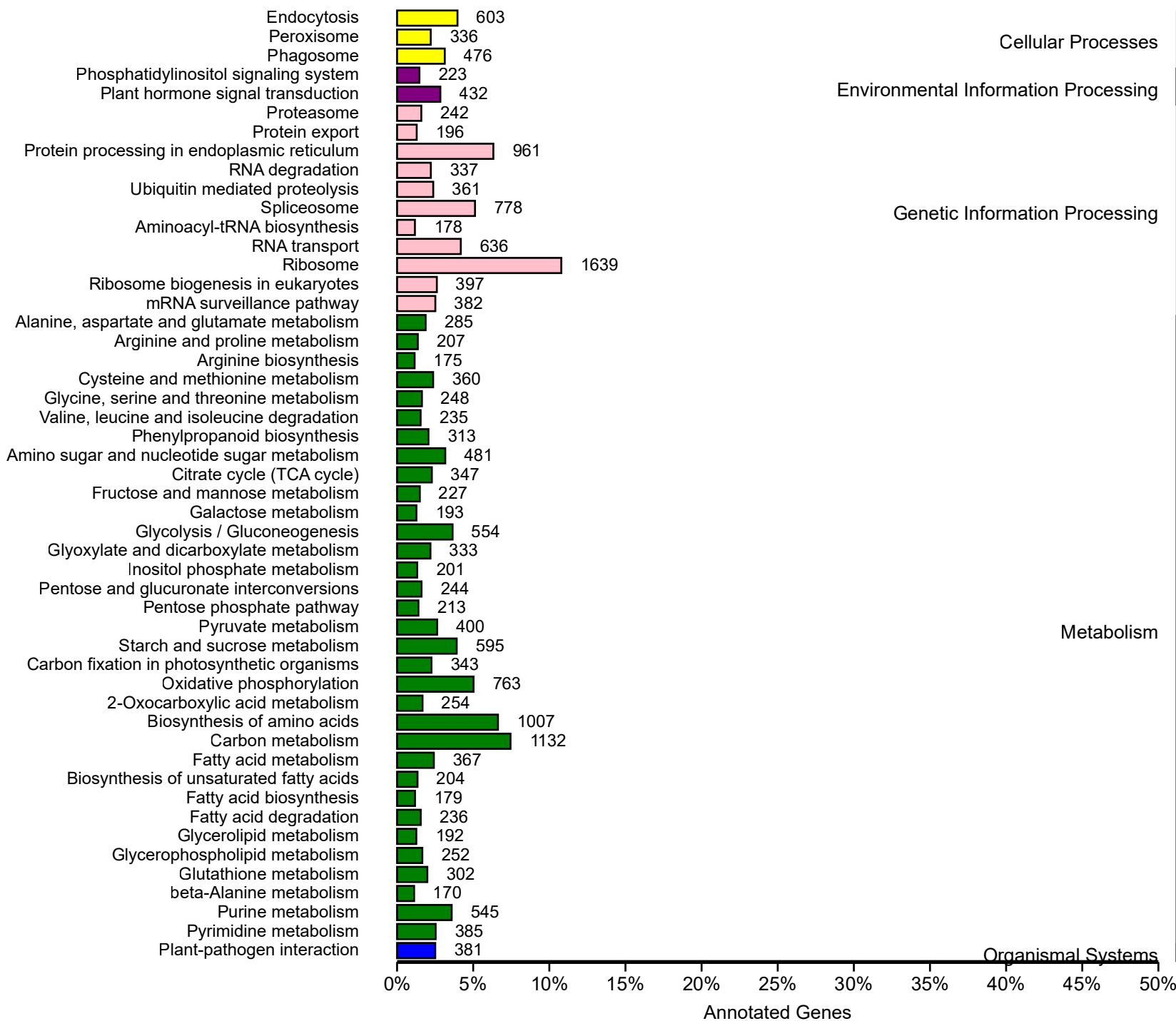

Supplement: Supplementary file 5 — Supplementary Material 5. [file 12864_2024_10142_MOESM5_ESM.pdf]

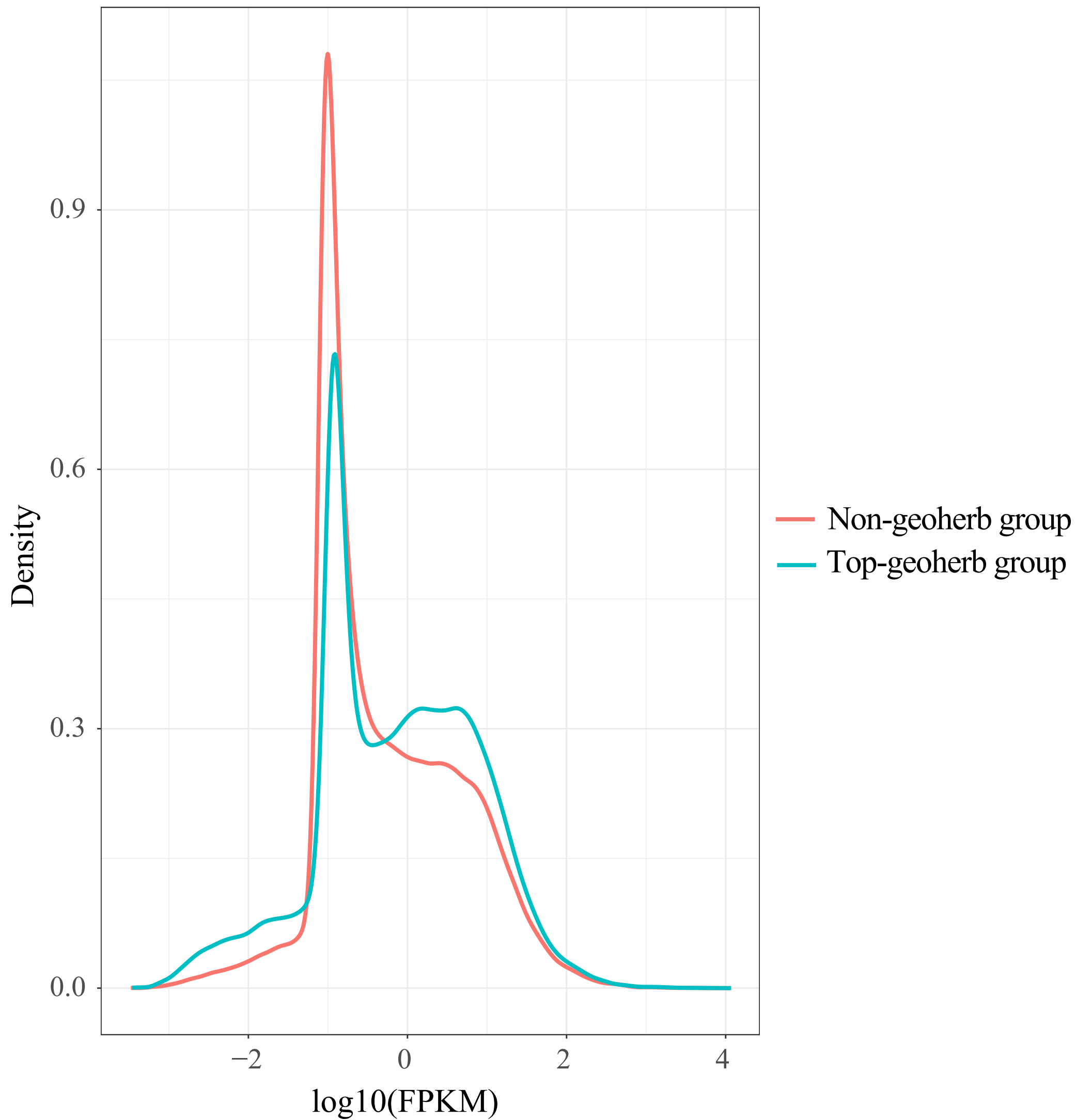

Supplement: Supplementary file 6 — Supplementary Material 6. [file 12864_2024_10142_MOESM6_ESM.pdf]

Statistics of Pathway Enrichment

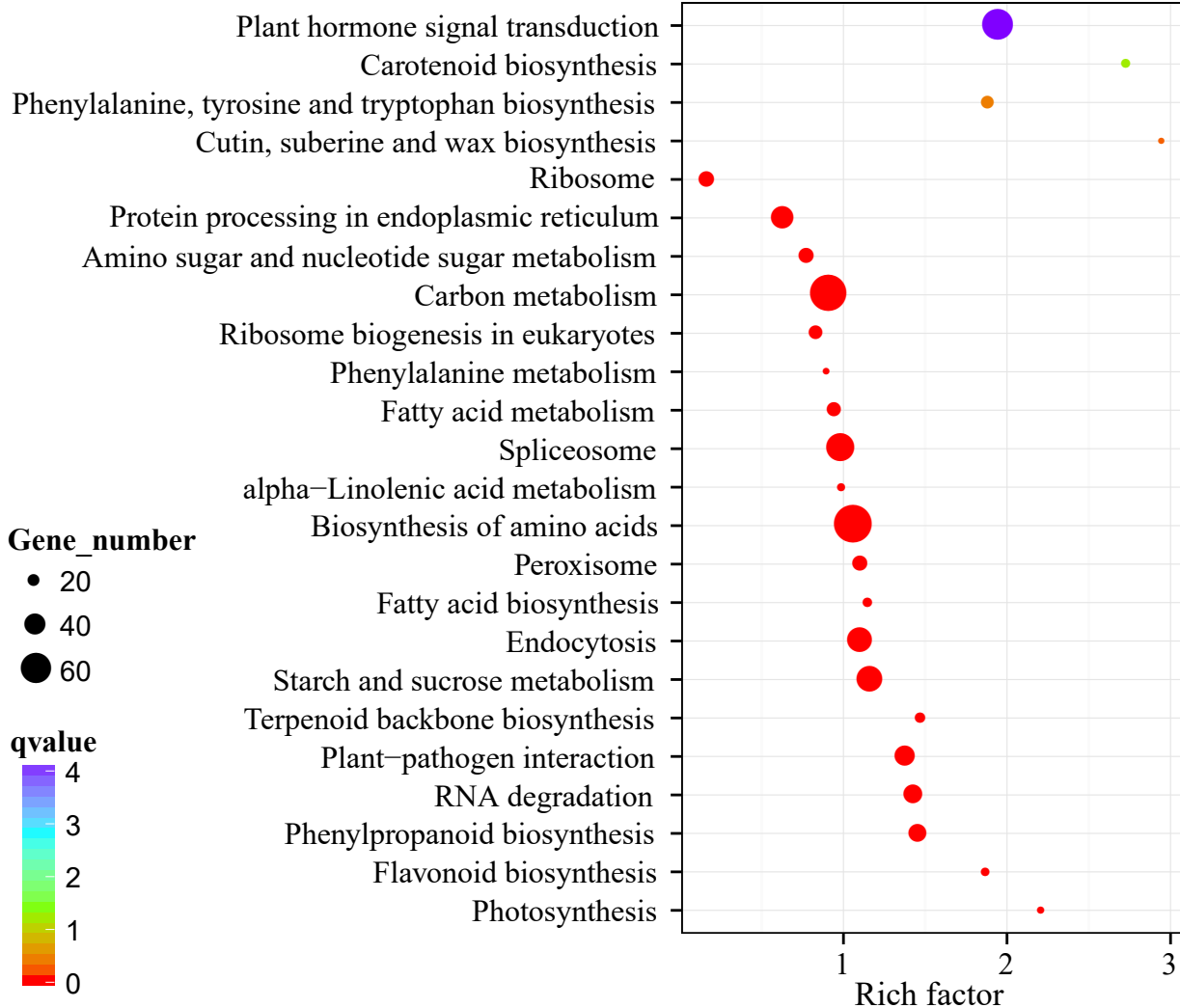

Supplement: Supplementary file 7 — Supplementary Material 7. [file 12864_2024_10142_MOESM7_ESM.pdf]

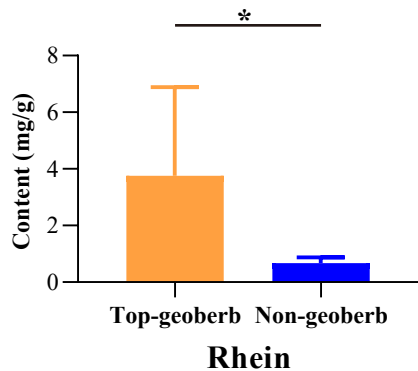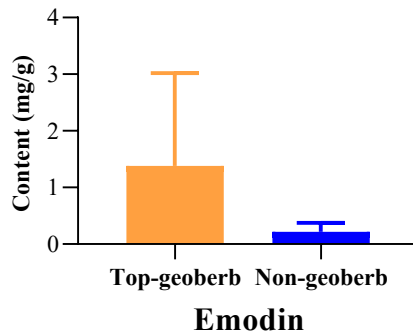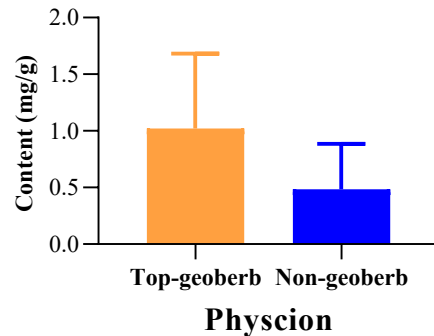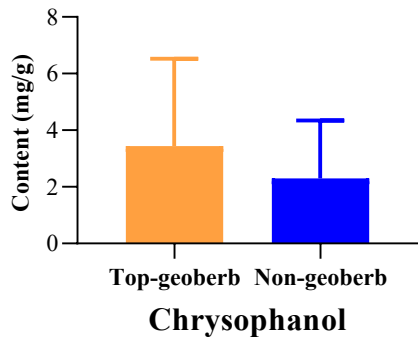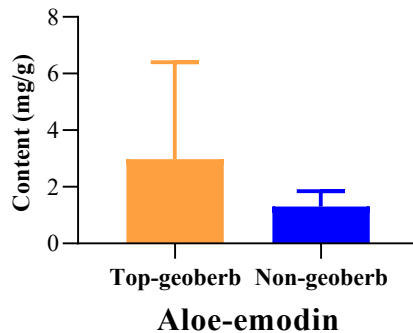

Supplement: Supplementary file 8 — Supplementary Material 8. [file 12864_2024_10142_MOESM8_ESM.pdf]

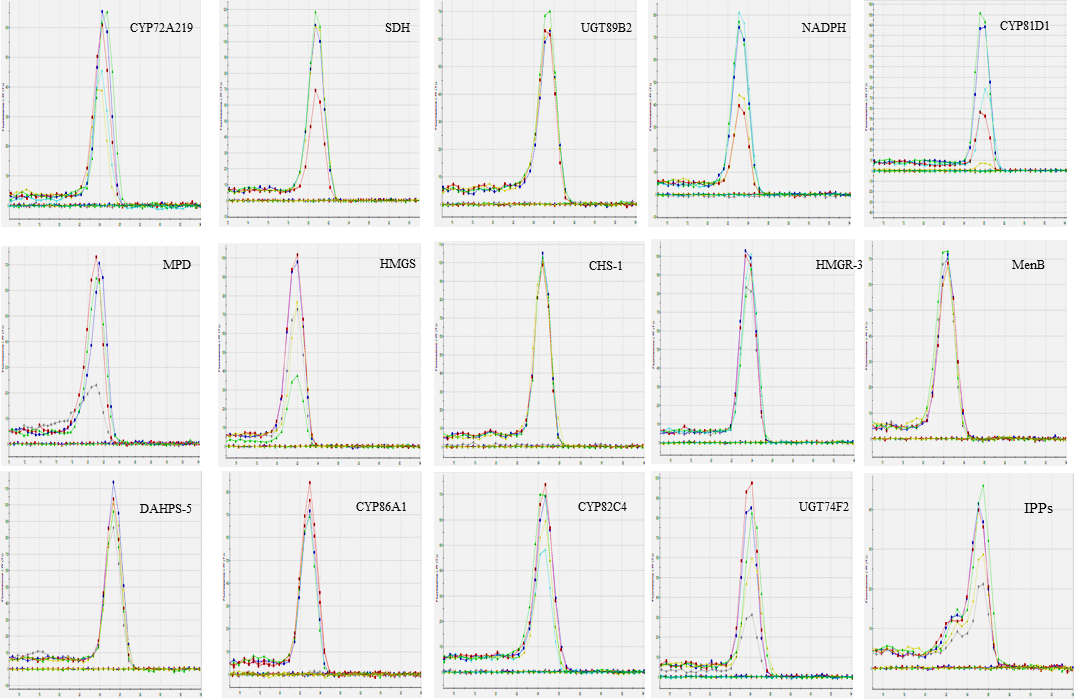

Supplement: Supplementary file 9 — Supplementary Material 9. [file 12864_2024_10142_MOESM9_ESM.tif]
