# Supplementary material for: Transcriptomic divergence of the Rheum palmatum complex derived from top-geoherb and non-geoherb areas provides the insights into geoherbalism properties of rhubarb
Source: BMC Genomics. 2024 Feb 26;25:212. doi: 10.1186/s12864-024-10142-3 (PMC10898026; doi:10.1186/s12864-024-10142-3)
Supplement: Supplementary file 4 — Supplementary Material 4. [file 12864_2024_10142_MOESM4_ESM.pdf]

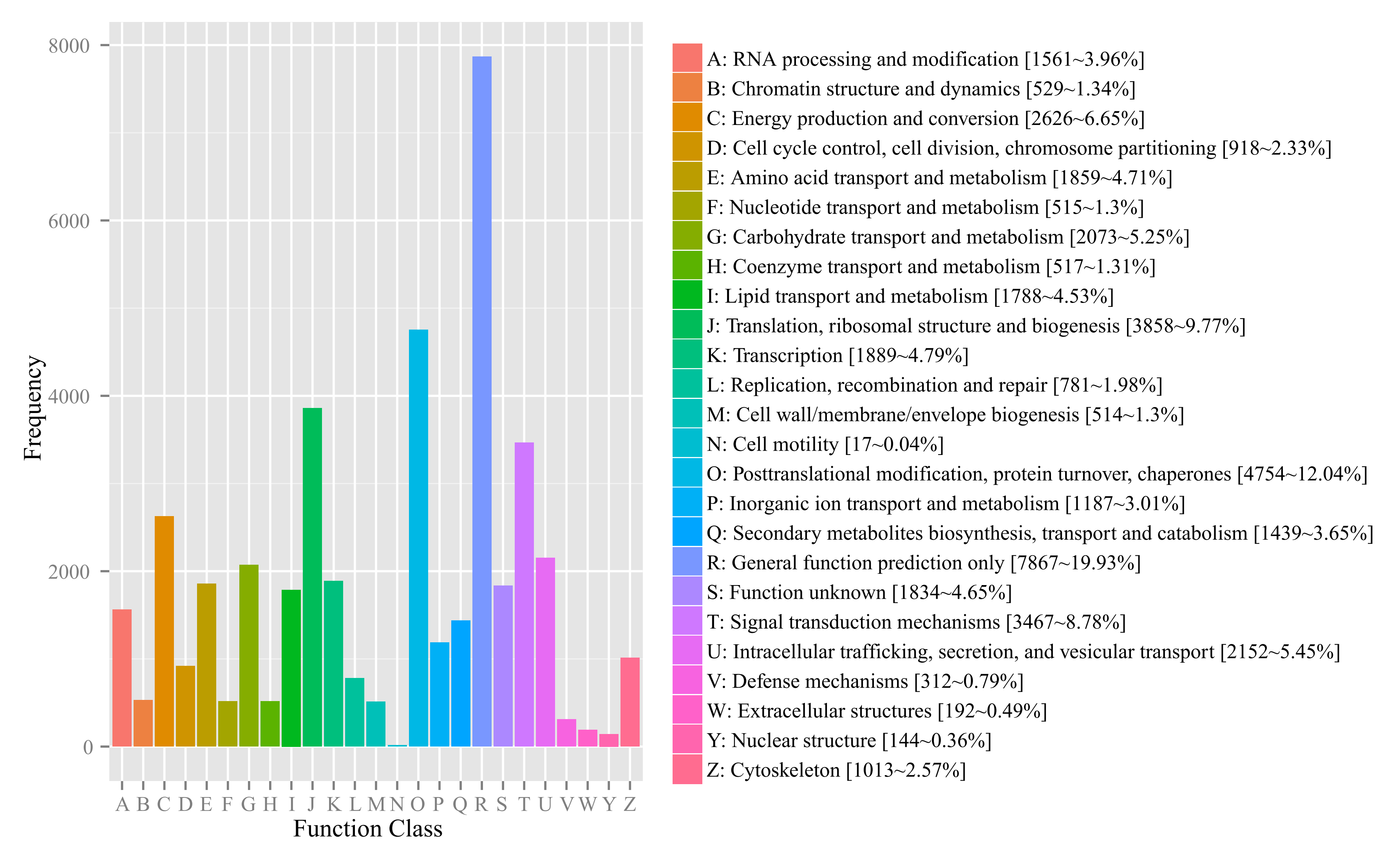
Frequency
8000
6000
4000
2000
0
A B C D E F G H I J K L M N O P Q R S T U V W Y Z
Function Class
A: RNA processing and modification [1561~3.96%]
B: Chromatin structure and dynamics [529~1.34%]
C: Energy production and conversion [2626~6.65%]
D: Cell cycle control, cell division, chromosome partitioning [918~2.33%]
E: Amino acid transport and metabolism [1859~4.71%]
F: Nucleotide transport and metabolism [515~1.3%]
G: Carbohydrate transport and metabolism [2073~5.25%]
H: Coenzyme transport and metabolism [517~1.31%]
I: Lipid transport and metabolism [1788~4.53%]
J: Translation, ribosomal structure and biogenesis [3858~9.77%]
K: Transcription [1889~4.79%]
L: Replication, recombination and repair [781~1.98%]
M: Cell wall/membrane/envelope biogenesis [514~1.3%]
N: Cell motility [17~0.04%]
O: Posttranslational modification, protein turnover, chaperones [4754~12.04%]
P: Inorganic ion transport and metabolism [1187~3.01%]
Q: Secondary metabolites biosynthesis, transport and catabolism [1439~3.65%]
R: General function prediction only [7867~19.93%]
S: Function unknown [1834~4.65%]
T: Signal transduction mechanisms [3467~8.78%]
U: Intracellular trafficking, secretion, and vesicular transport [2152~5.45%]
V: Defense mechanisms [312~0.79%]
W: Extracellular structures [192~0.49%]
Y: Nuclear structure [144~0.36%]
Z: Cytoskeleton [1013~2.57%]
